# Close Similarity between Sequences of Hepatitis E Virus Recovered from Humans and Swine, France, 2008−2009

**DOI:** 10.3201/eid1711.110616

**Published:** 2011-11

**Authors:** Jérôme Bouquet, Sophie Tessé, Aurélie Lunazzi, Marc Eloit, Nicolas Rose, Elisabeth Nicand, Nicole Pavio

**Affiliations:** Anses, Laboratoire de Santé Animale, Maisons-Alfort, France (J. Bouquet, A. Lunazzi, N. Pavio);; Hôpital des Armées Val de Grâce, Paris, France (S. Tessé, E. Nicand);; Ecole Nationale Vétérinaire d’Alfort, Maisons-Alfort (M. Eloit);; Anses, Laboratoire de Ploufragan-Plouzané, Ploufragan, France (N. Rose)

## Medscape CME

Medscape, LLC is pleased to provide online continuing medical education (CME) for this journal article, allowing clinicians the opportunity to earn CME credit.

This activity has been planned and implemented in accordance with the Essential Areas and policies of the Accreditation Council for Continuing Medical Education through the joint sponsorship of Medscape, LLC and Emerging Infectious Diseases. Medscape, LLC is accredited by the ACCME to provide continuing medical education for physicians.

Medscape, LLC designates this Journal-based CME activity for a maximum of 1 *AMA PRA Category 1 Credit(s)*^TM^. Physicians should claim only the credit commensurate with the extent of their participation in the activity.

All other clinicians completing this activity will be issued a certificate of participation. To participate in this journal CME activity: (1) review the learning objectives and author disclosures; (2) study the education content; (3) take the post-test with a 70% minimum passing score and complete the evaluation at www.medscape.org/journal/eid; (4) view/print certificate.

**Release date: October 21, 2011; Expiration date: October 21, 2012**

## Learning Objectives

Upon completion of this activity, participants will be able to:

- Describe epidemiologic features of autochthonous hepatitis E virus (HEV) infections based on a French study
- Compare the genetic identity of HEV strains found in humans and swine during an 18-month period in France
- Describe the public health implications of these findings.

## EDITOR

**Beverly D. Merritt,** Technical Writer/Editor, *Emerging Infectious Diseases. Disclosure: Beverly D. Merritt has disclosed no relevant financial relationships*.

## Medscape CME AUTHOR

**Laurie Barclay, MD,** freelance writer and reviewer, Medscape, LLC. *Disclosure: Laurie Barclay, MD, has disclosed no relevant financial relationships.*

## AUTHORS

*Disclosures:* ***Jérôme Bouquet; Sophie Tessé, MD; Aurélie Lunazzi; Nicolas Rose; Elisabeth Nicand, MD;*** *and* ***Nicole Pavio*** *have disclosed no relevant financial relationships.* ***Marc Eloit*** *has disclosed the following relevant financial relationships: served as an advisor or consultant for Sanofi-Aventis, GlaxoSmithKline, LEB, Genevrien, Pierre Fabre, Solvat; owns stock, stock options, or bonds from Vivalis; employed by Pathoquest.*
