# Supplementary material for: Close Similarity between Sequences of Hepatitis E Virus Recovered from Humans and Swine, France, 2008−2009
Source: Emerg Infect Dis. 2011 Nov;17(11):2018–25. doi: 10.3201/eid1711.110616 (PMC3311115; doi:10.3201/eid1711.110616)
Supplement: Related texts

# Research article: Close Similarity between Sequences of Hepatitis E Virus Recovered from Humans and Swine, France, 2008−2009

**DOI:** 10.3201/eid1711.110616

**Authors:** Jérôme Bouquet, Sophie Tessé, Aurélie Lunazzi, Marc Eloit, Nicolas Rose, Elisabeth Nicand, Nicole Pavio

**Affiliations:** Anses, Laboratoire de Santé Animale, Maisons-Alfort, France (J. Bouquet, A. Lunazzi, N. Pavio);; Hôpital des Armées Val de Grâce, Paris, France (S. Tessé, E. Nicand);; Ecole Nationale Vétérinaire d’Alfort, Maisons-Alfort (M. Eloit);; Anses, Laboratoire de Ploufragan-Plouzané, Ploufragan, France (N. Rose)

## Abstract

Frequent zoonotic transmission of hepatitis E virus (HEV) has been suspected, but data supporting the animal origin of autochthonous cases are still sparse. We assessed the genetic identity of HEV strains found in humans and swine during an 18-month period in France. HEV sequences identified in patients with autochthonous hepatitis E infection (n = 106) were compared with sequences amplified from swine livers collected in slaughterhouses (n = 43). Phylogenetic analysis showed the same proportions of subtypes 3f (73.8%), 3c (13.4%), and 3e (4.7%) in human and swine populations. Furthermore, similarity of >99% was found between HEV sequences of human and swine origins. These results indicate that consumption of some pork products, such as raw liver, is a major source of exposure for autochthonous HEV infection.

Hepatitis E virus (HEV) is a causative agent of enterically transmitted acute hepatitis in humans (*1*). It is a major public health issue in developing countries, where it causes large waterborne epidemics (*2*). In industrialized countries, it is an emerging problem, as an increasing number of sporadic cases for which the origins are still unclear (*3*) have been reported for patients who have not traveled to HEV-endemic areas.

HEV is a nonenveloped virus with a single-stranded positive RNA genome of 7.2 kb composed of 3 open reading frames (ORFs). HEV is the sole member of the family *Hepeviridae* (*4*) and has been classified into 4 major genotypes and 24 subtypes. Genotype 1 is divided into 5 subtypes (1a to 1e), genotype 2 into 2 subtypes (2a and 2b), genotype 3 into 10 subtypes (3a to 3j), and genotype 4 into 7 subtypes (4a to 4g) (*5*). Although genotypes 1 and 2 are endemic to developing countries, genotypes 3 and 4 are the cause of sporadic cases. HEV is the only hepatitis virus that is also found in a wide variety of animals (*6*). Genotype 3 can infect humans as well as swine, wild boar, deer, and mongoose (*7**–**10*). It is generally agreed that swine are widely infected all over the world (*6*). HEV seroprevalence varies greatly depending on countries; 22.7% to 88.4% of pigs are seropositive at 6 months of age (*11**,**12*). Among pigs slaughtered at ≈25 weeks of age, the prevalence of HEV fecal excretion ranges from 4% to 41% (*13**,**14*). Viral RNA sequences from pigs and humans can be closely related (*15**,**16*), and cross-species infection of genotypes 3 and 4 from human to pig and pig to nonhuman primate has been demonstrated experimentally (*17*). To date, only 2 cases of zoonotic transmission from consumption of raw or undercooked sika deer and wild boar meat have been clearly identified in Japan with near or 100% homology between the sequences from the patient and the consumed meat (*7**,**8*).

A few reports have shown close phylogenetic relationships between sequences identified in swine and in humans. However, these studies were based on limited numbers of sequences with little geographic or temporal data (*18**–**21*).

In France, HEV seroprevalence in the human population ranges from 3.2% to 16.6%, depending on the geographic regions studied (*22**,**23*). The number of reported viral hepatitis E cases is increasing. Although only 38 cases were reported in 2006, a total of 340 cases were diagnosed in 2010, of which 70% were declared autochthonous with no recent history of patients traveling abroad (French National Reference Laboratory, unpub. data). In the swine reservoir, a recent nationwide survey performed at slaughterhouses showed high prevalence of HEV. HEV seroprevalence in swine ranges from 31% at the individual level to 65% at the farm level. In that study, HEV prevalence in pig liver was estimated at 4%, meaning that HEV-infected pig livers can enter the food chain (*24*). Moreover, it has been shown that regional products made from raw pig liver may contain HEV (*25*). In France, pork is the most widely eaten type of meat (*26*) and could represent an HEV reservoir with a high risk for zoonotic transmission.

To assess the zoonotic risk for transmission from swine to humans in France, we studied HEV sequences in both hosts. HEV sequences collected from every human autochthonous case of hepatitis E infection and HEV-positive pig livers collected at slaughterhouses, both within 18 months, were analyzed. Epidemiologic and spatial–temporal data corresponding to phylogenetic analyses of partial ORF2 sequences were used to investigate whether swine are a major source of HEV contamination in France.

## Materials and Methods

### HEV Patients

Persons who had autochthonous hepatitis E virus infection during May 2008–November 2009 and had no travel history outside France were included in the study. RNA was extracted from patient serum or fecal samples by using a MagNA Pure LC RNA Isolation Kit (MagNA Pure LC Instrument; Roche Diagnostics, Basel, Switzerland) according to the manufacturer’s instructions. HEV RNA was amplified by using a nested reverse transcription PCR for the ORF2 gene as described (*27*). Sequencing was performed on amplified strands with an automated DNA sequencer (CEQ8000; Beckman-Coulter Inc., Fullerton, CA, USA). Patients’ demographic and epidemiologic features were collected anonymously from a questionnaire on age, sex, recent travel (within the past 4 months), and medical history.

### Swine Sample Collection

As part of a national survey on the prevalence of swine infected with HEV, 3,715 liver samples were collected at slaughterhouses from May 2008 through November 2009. Pig farms were selected through random sampling from 35 slaughterhouses accounting for 95% of the national pig production. Herds were selected randomly from a database table indicating dates and times of slaughter regardless of the herd size, leading to a random distribution of small and large types of farms (*24*). Thirty milligrams of liver was excised with sterile surgical blades. Tissues were disrupted in bead-milling tubes (FastPrep 24; MP Biomedicals, Illkrish, France). RNA was extracted by using the RNeasy Viral RNA extraction kit (QIAGEN, Courtaboeuf, France) according to the manufacturer’s instructions.

HEV RNA was detected by nested reverse transcription PCR with the same primers used for human HEV amplification (*27*). Positive samples were sequenced by the Sanger method (Cogenics, Grenoble, France or Eurofins MWG Operon, Ebersberg, Germany).

### Phylogenetic Analysis

We deposited 106 HEV sequences from human patients (1 sequence/patient) in GenBank under accession nos. JF730329–JF730434 and 43 HEV sequences from swine livers (1 sequence/farm) under accession nos. JF718787–JF718829. Human and swine HEV RNA sequences of 204 to 306 nt were analyzed by using MEGA4 (*28*), with a set of sequences available from GenBank (Table A1), to determine genotypes and subtypes as described by Lu et al. (*5*). Alignment was performed by using ClustalW (MEGA4, www.megasoftware.net). Phylogenetic trees were built by using the neighbor-joining method with a bootstrap of 1,000 replicates.

### Statistical Analyses

Statistical analyses were performed by using a χ^2^ distribution with 1 df and the Fisher exact probability test to compare proportions between the 2 groups. Differences were considered to be statistically significant when p<0.05.

## Results

### Epidemiologic Data

During May 2008–November 2009, hepatitis E was diagnosed for 305 patients in France. Only the 106 patients who had answered and returned the questionnaire and who had no recent history of traveling abroad were included in the study.

Of the 106 patients with HEV viremia, information on sex and age was available for 103 patients, of whom 72% were men; the mean age was 55 years (Figure 1). The 40–69-year age group had a significantly predominant number of male patients (81%). All patients had acute resolving hepatitis E, except for 1 in whom chronic hepatitis E developed after a liver transplant.

**Figure 1:** Distribution of age and gender for 103 hepatitis E virus (HEV) viremic patients, France, May 2008–November 2009.

### Geographic Distribution of Human Cases and HEV-positive Swine Herds

Geographic data on place of residence were available for 100 patients. Most human HEV cases were diagnosed in southern France (67%), especially in the southeastern region, Provence-Alpes-Côte-d’Azur, which accounted for 30% of the cases (Figure 2) and contains 7.6% of the national population. In northern France, where 33% of the cases were observed, a high density of HEV cases (11%) were clustered in the Paris region (Figure 2). The Paris area, Ile-de-France, is the most populated region and contains 18% of the total population (*29**)*. In contrast, most of the HEV-positive swine herds were found in northern France (77%), particularly in the western region, Brittany, which is the largest swine-producing region, accounting for 52% of national production (Figure 2). Fewer positive swine samples were found in southern France (23%), where there is a lower density of pig herds than in Brittany (*24*).

**Figure 2:** Geographic distribution of hepatitis E virus (HEV) subtypes recovered from humans (n = 100) and swine (n = 43), France, May 2008–November 2009. Black, human HEVs; red, swine HEVs; triangles, subtype 3c; squares, subtype 3e; dots, subtype 3f; diamonds, strains of undefined subtype. Regions with a high density of HEV are named.

### Human and Swine HEV Sequences

To characterize HEV circulating in humans and swine from May 2008 through November 2009, we subjected partial ORF2 HEV sequences, amplified for both populations, to phylogenetic analysis. This ORF2 genomic region seems to match the classification of full-length HEV sequences according to Lu et al. (*5*) and gives similar phylogenetic topologies to the ORF1 region RdRp (*30*). For each human case, a single HEV sequence was retrieved (n = 106). One HEV RNA sequence from each positive farm was included when the same sequence was recovered from several pig livers from the same farm (n = 43). To define genotypes and subtypes, we added 22 reference sequences of human and swine origins to the analysis (Table A1). Genotype 4 HEV was used as the outgroup.

Human and swine strains were scattered homogeneously on the phylogenetic tree (Figure 3); no specific cluster in relation to the host was considered. All sequences belonged to genotype 3 and more specifically to subtypes 3f, 3c, and 3e. There was some difficulty in identifying a specific subtype to a cluster of 12 sequences, 8 from humans and 4 from swine. These sequences were close to 7 subtypes (3a, 3b, 3c, 3d, 3h, 3i, and 3j) but shared <90% homology with any of them (*5*). The term undefined subtype was given to this cluster (Figure 3).

**Figure 3:** Phylogenetic tree of hepatitis E virus (HEV) detected in human and swine constructed by the neighbor-joining method with a bootstrap of 1,000 replicates based on the ClustalW alignment (MEGA4, www.megasoftware.net) of 204- to 306-nt sequences within open reading frame 2. The 106 HEV sequences recovered from patients from France are displayed as black dots (GenBank accession nos. JF730329−JF730434), the 43 HEV sequences recovered from swine from France are displayed as red dots (accession nos. JF718787−JF718829), and the 22 reference strains from GenBank are displayed as white dots (GenBank accession nos. in Table A1). Genotype 4, subtypes 3f, 3c, and 3e, as defined by Lu et al. (*8*), are encircled by a solid black line; undefined subtype is encircled by a dashed black line. Bootstrap values >70% are indicated on respective branches. Scale bar represents nucleotide substitutions per site.

### Subtype Proportions and Distribution

A comparison of subtype proportions in swine and human populations did not reveal any significant differences (p>0.05) (Table 1). Subtype 3f was the largest cluster, accounting for 73.8% of the strains sequenced (72.6% in humans and 76.7% in swine). Subtype 3c was the second largest group, accounting for 13.4% of HEV strains (15.1% in humans and 9.3% in swine). The set of sequences of undefined subtype accounted for 8.1% of the total strains and was also homogeneously represented (no statistical difference in proportion) between human (7.6%) and swine strains (9.3%). Finally, the proportion of subtype 3e was smallest, 4.7% in the swine and the human groups.

**Table 1:** Proportions of subtypes of HEV strains identified in 106 humans and 43 swine, France, May 2008–November 2009*

Sequences	No. (%) isolates				Total no.
	Subtype 3c	Subtype 3e	Subtype 3f	Undefined subtype	
Human HEV	16 (15.1)	5 (4.7)	77 (72.6)	8 (7.6)	106
Swine HEV	4 (9.3)	2 (4.7)	33 (76.7)	4 (9.3)	43
Total HEV	20 (13.4)	7 (4.7)	110 (73.8)	12 (8.1)	149

*HEV, hepatitis E virus.

Geographic distribution of subtypes showed that 3f was found all over the territory; 3c seemed to be missing in Brittany, where the largest number of samples was collected (1,760 livers). Most sequences of the undefined subtype originated from southern France (Figure 2).

### Nucleotide Variations among Human and Swine HEV Sequences

To investigate whether some nucleotide positions would be host strain specific, a p-value was calculated for each nucleotide position. No significant difference (p<0.05) between human and swine HEV was obtained for the short nucleotide sequence studied (data not shown). The same observation was made at the amino acid level, where there was no significant difference at any position between human and swine HEV (data not shown).

### HEV Sequence Similarities

#### Human Sequence Similarities

The 106 sequences recovered from human patients were compared with each other at the nucleotide level. The percentage of nucleotide sequence identities ranged from 67.8% to 100% (Table 2). Four groups of 2–3 patients had 100% nt similarity. These sequences were detected in patients living in different regions, at intervals ranging from 6 days to 6 months (Figure 4).

**Table 2:** Percentage nucleotide similarities of 204 to 306 HEV nucleotide sequences from 106 humans and 43 swine, France, May 2008–November 2009

Sequences	Minimum	Average	Median	Maximum
Human HEV	67.8	85.2	87.7	100
Swine HEV				
Same farm	99.0	99.7	99.7	100
Different farms	71.7	86.1	88.2	99.6
Human and swine HEV	68.4	85.4	87.6	99.3

*HEV, hepatitis E virus.

**Figure 4:** Geographic distribution and sampling date of human and swine hepatitis E virus (HEV) sequences sharing >99% identities, France, May 2008–November 2009.

#### Swine Sequence Similarities

First, to evaluate HEV within-farm homology, we compared 10 sequences recovered on the same day from 10 animals from the same farm. Similarities of 99% to 100% were found (Table 2). The 43 sequences recovered from independent farms all over France were then compared with each other. Similarities ranged from 71.7% to 99.3% (Table 2). Three pairs of sequences were found with similarities of >99% (Figure 4). These pairs of sequences originated from neighboring farms (Figure 4). Two pairs of sequences were sampled on the same day (August 30, 2008 or November 18, 2009), and the third pair was sampled at a 6-month interval (June 10, 2008, and November 24, 2008).

#### Similarities between Human and Swine Sequences

Similarities ranged from 68.4% to 99.3% (Table 2). These minimum and maximum similarities do not significantly differ from those found in each separate population (p<0.05).

Two pairs of sequences were found to have >99% similarity. In both cases, human and animal HEV sequences were identified in different geographic regions at intervals of 5 months (human, August 3, 2008; and swine, April 9, 2008) to 1 year (human, May 22, 2009; and swine, May 27, 2008). In both cases, swine sequences were sampled first, before the onset of the disease in the patient.

## Discussion

Although zoonotic transmission of hepatitis E virus from swine to human has been well accepted, little data are available on HEV sequences circulating in human and swine populations within a country during a restricted period. We investigated a large number of HEV sequences, collected from 106 patients and 43 swine over an 18-month period. The patients were mostly male (72%) and >55 years of age. This finding is in agreement with a previous report on acute HEV infection in France, which found that men accounted for 68% (36/53) of the cases (*31*). The situation in industrialized countries contrasts with that in regions where attack rates for waterborne outbreaks of HEV genotype 1 are higher among young adults (15–40 years of age) (*1*). This observation suggests that the 2 epidemiologic profiles may involve different contamination routes. There are differences in hygiene and meat consumption habits in these regions. Moreover, no animal reservoirs have been yet described for the genotypes involved in waterborne outbreaks (genotypes 1 or 2) (*6*), suggesting that zoonotic transmission might be limited in any HEV-endemic areas.

All 149 HEV sequences belonged to genotype 3 and were divided into at least 3 subtypes according to the classification elaborated by Lu et al. (*5*). Sequences sharing a minimum of 90% similarity were considered as belonging to the same subtype. Among these sequences, 137 belonged to subtypes 3f, 3c, and 3e. For 12 sequences, 8 human HEV and 4 swine HEV, there was some classification uncertainty because they were close to 7 different subtypes but shared <90% homology. The difficulty in classifying this undefined subtype might be because of partial sequencing of the strains identified, although Lu et al. showed that the 5′ end of the ORF2 region matches the complete genomic sequence for HEV classification better than other regions of the HEV genome (*5*). Using the nucleotide BLAST database (http://blast.ncbi.nlm.nih.gov/Blast), sequences from this undefined subtype are close but share <90% homology to 3a and 3c sequences detected in the Netherlands or 3h and 3i sequences detected in Germany. This undefined subtype also clusters on its own (>90% homology) and could be a new subtype that is specific to France. Comparison of autochthonous HEV from France with HEV from neighboring countries shows that the same main subtypes are found: 3f is found all over Europe; 3c in the Netherlands, Italy, and Hungary; and 3e in the UK, the Netherlands, Germany, and Hungary (*15**,**18**,**32**–**35*). This finding suggests that some subtypes may have emerged and evolved locally through animal trading.

The proportion of each subtype in both species was then estimated, and the proportions of subtypes 3f, 3c, and 3e were found to be almost the same. Such a similar distribution of subtypes suggests an active circulation of the virus between the 2 host species in France. In the Netherlands, proportions of subtypes in human compared with animals or environmental strains were found to differ markedly, 6% versus 43% for 3f and 75% versus 35% for 3c (*18*), suggesting a limited number of contamination events through these 2 possible contamination pathways in this country.

Although HEV is widely distributed across France, some geographic regions showed higher rates of infection in humans. Most (67%) cases of autochthonous hepatitis E were found in southern France and particularly in the Provence-Alpes-Côte-d’Azur region (30%). These results are consistent with HEV seroprevalence in blood donors being higher in southern (16.6%) than in northern France (3.2%) (*22**,**23*). Furthermore, this observation correlates with results of a previous national survey in France showing an increasing north-to-south gradient of acute hepatitis E (*31*). In contrast, in the animal reservoir, most HEV sequences were detected in the main pig-producing area located in northwestern France. Nevertheless, the low number (only 2) of human cases observed in this region with a high density of pig farms suggests that the number of contamination events through the environmental pathway is limited. In the Ile de France region (Paris area), a high number (11%) of cases of hepatitis E was also reported. This finding could be partially explained by the high population density (18%) in this area; a few cases were reported after traveling and eating uncooked pork products in southern France.

To further analyze sequence similarities between human and swine HEV strains, we determined the similarities in nucleotides between human and swine sequences. HEV has a high mutation rate because of its error-prone RNA-dependent RNA polymerase and is probably present as a quasispecies in an infected host (*36*). Thus, low (<1%) variability in nucleotides may correspond to a unique strain. Analyzing human HEV sequences, 100% nt similarity was found in 4 groups of 2–3 patients. These patients were not related, but they may have been exposed to an unknown common source of contamination.

Swine sequences amplified from livers of animals within the same herd were found to be homogeneous, with a maximum difference of 3 nt along the 306 nt sequenced. Except for 3 groups of 2 herds, all the sequences were different (<99%). These 3 groups included herds that were sampled at the same time or 5 months apart and that were geographically close (a few kilometers). This high similarity of partial sequences might be explained by a possible exchange of animals between nearby herds, which is a common practice. However, it cannot be excluded that movements of farm workers and veterinarians or spreading of infected slurry might contribute to HEV transmission between herds. Swine HEV infection spreads easily within a herd through the fecal–oral route (*37*). This geographic clustering of HEV strains detected in animals was also observed in Sweden (*19*).

Because animals from the same herd can have a difference of 3 nt over the same amplified sequence, human and porcine HEV sequences with >99% similarities may be considered as coming from related strains. A comparison of human and swine sequences showed that 2 pairs of sequences were similar (99.3%). In both cases, swine and human sequences were detected in different geographic areas. The swine sequences were identified first and later in humans. Pork meat is the most widely eaten meat in France (34.7 kg/inhabitant/year), and it is distributed and consumed throughout France (*26*). In our study, HEV sequences were amplified from liver, but other meat might be a vector for HEV infection because it has been shown that other organs such as muscles can be HEV positive (*38*). Considering the geographic distances and the detection of these HEV sequences in animals first, it seems reasonable to assume that foodborne infection may play a major role in autochthonous cases of hepatitis E. The high similarity observed suggests that these 2 cases could be the result of zoonotic transmission. Furthermore, because since these sequences are not geographically linked, contamination through environmental exposure can be ruled out.

In addition to the high degree of similarity observed between human and swine sequences and the identical proportion of each subtype in both hosts, no specific nucleotide substitutions have been identified when sequences from different host species were compared. These results are in line with the possible absence of a species barrier for HEV strains of genotype 3. However, before concluding that there are no host restriction determinants, further analysis of longer sequences is required.

This unique large-scale study on human and swine sequences with spatial–temporal data suggests that zoonotic transmission of HEV is involved in autochthonous cases. The swine reservoir is widely infected with HEV, and infected livers enter the food chain. Living in southern France seems to be associated with more frequent exposure to HEV (67% of cases). This observation might be linked to cultural food habits specific to southern France and frequent consumption of products made from raw swine liver (*25*).

Slurry from swine is often spread onto local fields, but there are few (only 2) cases reported in Brittany compared with other regions. The spread of HEV into the environment may not have major consequences but cannot be ignored. Contact with animals; consumption of contaminated water, vegetables, or shellfish; or unknown routes of transmission need to be investigated. In conclusion, taken together, these results confirm the major role played by the swine reservoir of HEV in autochthonous cases of hepatitis E. This study underlines the need for a surveillance and control plan, either at the level of pig production or at the level of food processing, to limit human exposure to HEV through consumption of pork products.

## Appendix

**Table A1:** List of hepatitis E virus reference sequences from Genbank used for phylogenetic analyses of strains identified in France, May 2008–November 2009*

Accession no.	Name	Host	Country	Genotype
AF336291	NLSW22	Swine	Netherlands	3a
AF082843	swUS1	Swine	USA	3a
AF466665	UMC12B	Swine	USA	3a
AF060668	US-1	Human	USA	3a
AB115544	HE-JA23	Human	Japan	3b
AB189075	JYO-Hyo03L	Human	Japan	3b
AB073912	swJ570	Swine	Japan	3b
AF336298	NLSW105	Swine	Netherlands	3c
AF336290	NLSW20	Swine	Netherlands	3c
AF296167	TW3SW	Swine	Taiwan	3d
AF503512	P143/11/02	Swine	UK	3e
AY362357	UK8734	Human	UK	3e
AY032758	NLSW50	Swine	Netherlands	3f
EU495148	TLS25	Human	France	3f
AF195061	VH1	Human	Spain	3f
AF455784	Osh205	Swine	Kyrgyzstan	3g
FJ705359	wbGER27	Wild boar	Germany	3i
AY115488	Arkell	Swine	Canada	3j
AB108537	CCC220	Human	China	4
AB161717	JSM-Sap95	Human	Japan	4
FJ610232	swCH189	Swine	China	4
AB097811	swJ13-1	Swine	Japan	4

*HEV, hepatitis E virus.

## References

[1] Panda SK, Thakral D, Rehman S. Hepatitis E virus. Rev Med Virol. 2007;17:151–80. 10.1002/rmv.52217051624

[2] Purcell RH, Emerson SU. Hepatitis E: an emerging awareness of an old disease. J Hepatol. 2008;48:494–503. 10.1016/j.jhep.2007.12.00818192058

[3] Dalton HR, Bendall R, Ijaz S, Banks M. Hepatitis E: an emerging infection in developed countries. Lancet Infect Dis. 2008;8:698–709. 10.1016/S1473-3099(08)70255-X18992406

[4] Emerson SU, Anderson D, Arankalle A, Meng XJ, Purdy M, Schlauder GG, Virus taxonomy VIIIth report of the ICTV. Hepevirus; 2004; 851–3.

[5] Lu L, Li C, Hagedorn CH. Phylogenetic analysis of global hepatitis E virus sequences: genetic diversity, subtypes and zoonosis. Rev Med Virol. 2006;16:5–36. 10.1002/rmv.48216175650

[6] Pavio N, Meng X, Renou C. Zoonotic hepatitis E: animal reservoirs and emerging risks. Vet Res. 2010;41:46. 10.1051/vetres/201001820359452PMC2865210

[7] Li TC, Chijiwa K, Sera N, Ishibashi T, Etoh Y, Shinohara Y, Hepatitis E virus transmission from wild boar meat. Emerg Infect Dis. 2005;11:1958–60.1648549010.3201/eid1112.051041PMC3367655

[8] Tei S, Kitajima N, Takahashi K, Mishiro S. Zoonotic transmission of hepatitis E virus from deer to human beings. Lancet. 2003;362:371–3. 10.1016/S0140-6736(03)14025-112907011

[9] Meng XJ, Purcell RH, Halbur PG, Lehman JR, Webb DM, Tsareva TS, A novel virus in swine is closely related to the human hepatitis E virus. Proc Natl Acad Sci U S A. 1997;94:9860–5. 10.1073/pnas.94.18.98609275216PMC23282

[10] Nakamura M, Takahashi K, Taira K, Taira M, Ohno A, Sakugawa H, Hepatitis E virus infection in wild mongooses of Okinawa, Japan: demonstration of anti-HEV antibodies and a full-genome nucleotide sequence. Hepatol Res. 2006;34:137–40. 10.1016/j.hepres.2005.10.01016413822

[11] Munné MS, Vladimirsky S, Otegui L, Castro R, Brajterman L, Soto S, Identification of the first strain of swine hepatitis E virus in South America and prevalence of anti-HEV antibodies in swine in Argentina. J Med Virol. 2006;78:1579–83. 10.1002/jmv.2074117063523

[12] dos Santos DRL, Vitral CL, de Paula VS, Marchevsky RS, Lopes JF, Gaspar AMC, Serological and molecular evidence of hepatitis E virus in swine in Brazil. Vet J. 2009;182:474–80. 10.1016/j.tvjl.2008.08.00118805029

[13] Leblanc D, Ward P, Gagné M, Poitras E, Müller P, Trottier Y, Presence of hepatitis E virus in a naturally infected swine herd from nursery to slaughter. Int J Food Microbiol. 2007;117:160–6. 10.1016/j.ijfoodmicro.2007.03.00817459506

[14] McCreary C, Martelli F, Grierson S, Ostanello F, Nevel A, Banks M. Excretion of hepatitis E virus by pigs of different ages and its presence in slurry stores in the United Kingdom. Vet Rec. 2008;163:261–5. 10.1136/vr.163.9.26118757902

[15] Banks M, Bendall R, Grierson S, Heath G, Mitchell J, Dalton H. Human and porcine hepatitis E virus strains, United Kingdom. Emerg Infect Dis. 2004;10:953–5.1520084110.3201/eid1005.030908PMC3323225

[16] van der Poel WH, Verschoor F, van der Heide R, Herrera MI, Vivo A, Kooreman M, Hepatitis E virus sequences in swine related to sequences in humans, the Netherlands. Emerg Infect Dis. 2001;7:970–6. 10.3201/eid0706.01060811747723PMC2631914

[17] Meng XJ, Halbur PG, Shapiro MS, Govindarajan S, Bruna JD, Mushahwar IK, Genetic and experimental evidence for cross-species infection by swine hepatitis E virus. J Virol. 1998;72:9714–21.981170510.1128/jvi.72.12.9714-9721.1998PMC110481

[18] Rutjes SA, Lodder WJ, Lodder-Verschoor F, van den Berg HHJL, Vennema H, Duizer E, Sources of hepatitis E virus genotype 3 in the Netherlands. Emerg Infect Dis. 2009;15:381–7. 10.3201/eid1503.07147219239749PMC2681103

[19] Widén F, Sundqvist L, Matyi-Toth A, Metreveli G, Belák S, Hallgren G, Molecular epidemiology of hepatitis E virus in humans, pigs and wild boars in Sweden. Epidemiol Infect. 2011;139:361–71. 10.1017/S095026881000134220546635

[20] Reuter G, Fodor D, Forgách P, Kátai A, Szucs G. Characterization and zoonotic potential of endemic hepatitis E virus (HEV) strains in humans and animals in Hungary. J Clin Virol. 2009;44:277–81. 10.1016/j.jcv.2009.01.00819217346

[21] Zhang W, Yang S, Ren L, Shen Q, Cui L, Fan K, Hepatitis E virus infection in central China reveals no evidence of cross-species transmission between human and swine in this area. PLoS ONE. 2009;4:e8156. 10.1371/journal.pone.000815619997619PMC2785466

[22] Boutrouille A, Bakkali-Kassimi L, Crucière C, Pavio N. Prevalence of anti-hepatitis E virus antibodies in French blood donors. J Clin Microbiol. 2007;45:2009–10. 10.1128/JCM.00235-0717460057PMC1933073

[23] Mansuy JM, Legrand-Abravanel F, Calot JP, Peron JM, Alric L, Agudo S, High prevalence of anti-hepatitis E virus antibodies in blood donors from South West France. J Med Virol. 2008;80:289–93. 10.1002/jmv.2105618098159

[24] Rose N, Lunazzi A, Dorenlor V, Merbah T, Eono F, Eloit M, High prevalence of hepatitis E virus in French domestic pigs. Comp Immunol Microbiol Infect Dis. 2001;34:419–27. 10.1016/j.cimid.2011.07.00321872929

[25] Colson P, Borentain P, Queyriaux B, Kaba M, Moal V, Gallian P, Pig liver sausage as a source of hepatitis E virus transmission to humans. J Infect Dis. 2010;202:825–34. 10.1086/65589820695796

[26] Ministére de l’Agriculture et de la Péche. Consommation de viande en France. AGRESTE, 2008. 2008/n°29 [cited 2008 Jun]. http://www.agreste.agriculture.gouv.fr/IMG/pdf/syntheseviande0806.pdf

[27] Cooper K, Huang FF, Batista L, Rayo CD, Bezanilla JC, Toth TE, Identification of genotype 3 hepatitis E virus (HEV) in serum and fecal samples from pigs in Thailand and Mexico, where genotype 1 and 2 HEV strains are prevalent in the respective human populations. J Clin Microbiol. 2005;43:1684–8. 10.1128/JCM.43.4.1684-1688.200515814985PMC1081392

[28] Tamura K, Dudley J, Nei M, Kumar S. MEGA4: Molecular Evolutionary Genetics Analysis (MEGA) software version 4.0. Mol Biol Evol. 2007;24:1596–9. 10.1093/molbev/msm09217488738

[29] French Ministry of the Economy. Finance and Industry. Decree no. 2009-1707 of 30 December 2009. Paris: Official Journal of the French Republic, Direction de l’information légale et administrative; 2010.

[30] Legrand-Abravanel F, Mansuy J, Dubois M, Kamar N, Peron J, Rostaing L, Hepatitis E virus genotype 3 diversity, France. Emerg Infect Dis. 2009;15:110–4. 10.3201/eid1501.08029619116067PMC2660688

[31] Renou C, Moreau X, Pariente A, Cadranel J, Maringe E, Morin T, A national survey of acute hepatitis E in France. Aliment Pharmacol Ther. 2008;27:1086–93. 10.1111/j.1365-2036.2008.03679.x18346187

[32] Peralta B, Mateu E, Casas M, de Deus N, Martín M, Pina S. Genetic characterization of the complete coding regions of genotype 3 hepatitis E virus isolated from Spanish swine herds. Virus Res. 2009;139:111–6. 10.1016/j.virusres.2008.09.00818977254

[33] Adlhoch C, Wolf A, Meisel H, Kaiser M, Ellerbrok H, Pauli G. High HEV presence in four different wild boar populations in East and West Germany. Vet Microbiol. 2009;139:270–8. 10.1016/j.vetmic.2009.06.03219595519

[34] Di Bartolo I, Ponterio E, Castellini L, Ostanello F, Ruggeri FM. Viral and antibody HEV prevalence in swine at slaughterhouse in Italy. Vet Microbiol. 2011;149:330–8. 10.1016/j.vetmic.2010.12.00721216541

[35] Forgách P, Nowotny N, Erdélyi K, Boncz A, Zentai J, Szucs G, Detection of hepatitis E virus in samples of animal origin collected in Hungary. Vet Microbiol. 2010;143:106–16. 10.1016/j.vetmic.2009.11.00420005644

[36] Grandadam M, Tebbal S, Caron M, Siriwardana M, Larouze B, Koeck JL, Evidence for hepatitis E virus quasispecies. J Gen Virol. 2004;85:3189–94. 10.1099/vir.0.80248-015483231

[37] de Deus N, Casas M, Peralta B, Nofrarías M, Pina S, Martín M, Hepatitis E virus infection dynamics and organic distribution in naturally infected pigs in a farrow-to-finish farm. Vet Microbiol. 2008;132:19–28. 10.1016/j.vetmic.2008.04.03618562132

[38] Bouwknegt M, Rutjes SA, Reusken CB, Stockhofe-Zurwieden N, Frankena K, de Jong MCM, The course of hepatitis E virus infection in pigs after contact-infection and intravenous inoculation. BMC Vet Res. 2009;5:7. 10.1186/1746-6148-5-719193209PMC2647918

# Research article: Close Similarity between Sequences of Hepatitis E Virus Recovered from Humans and Swine, France, 2008−2009

**DOI:** 10.3201/eid1711.110616

**Authors:** Jérôme Bouquet, Sophie Tessé, Aurélie Lunazzi, Marc Eloit, Nicolas Rose, Elisabeth Nicand, Nicole Pavio

**Affiliations:** Anses, Laboratoire de Santé Animale, Maisons-Alfort, France (J. Bouquet, A. Lunazzi, N. Pavio);; Hôpital des Armées Val de Grâce, Paris, France (S. Tessé, E. Nicand);; Ecole Nationale Vétérinaire d’Alfort, Maisons-Alfort (M. Eloit);; Anses, Laboratoire de Ploufragan-Plouzané, Ploufragan, France (N. Rose)

## Earning Medscape CME Credit

To obtain credit, you should first read the journal article. After reading the article, you should be able to answer the following, related, multiple-choice questions. To complete the questions (with a minimum 70% passing score) and earn continuing medical education (CME) credit, please go to www.medscape.org/journal/eid. Credit cannot be obtained for tests completed on paper, although you may use the worksheet below to keep a record of your answers. You must be a registered user on Medscape.org. If you are not registered on Medscape.org, please click on the New Users: Free Registration link on the left hand side of the website to register. Only one answer is correct for each question. Once you successfully answer all post-test questions you will be able to view and/or print your certificate. For questions regarding the content of this activity, contact the accredited provider, CME@medscape.net. For technical assistance, contact CME@webmd.net. American Medical Association’s Physician’s Recognition Award (AMA PRA) credits are accepted in the US as evidence of participation in CME activities. For further information on this award, please refer to http://www.ama-assn.org/ama/pub/category/2922.html. The AMA has determined that physicians not licensed in the US who participate in this CME activity are eligible for *AMA PRA Category 1 Credits*™. Through agreements that the AMA has made with agencies in some countries, AMA PRA credit may be acceptable as evidence of participation in CME activities. If you are not licensed in the US, please complete the questions online, print the certificate and present it to your national medical association for review.Article Title: Close Similarity between Sequences of Hepatitis E Virus Recovered from Humans and Swine, France, 2008−2009Medscape CME Questions1. Based on the French study by Dr. Bouquet and colleagues, which of the following statements about epidemiologic features of autochthonous hepatitis E virus (HEV) infections is most likely correct?A. Most cases are in womenB. Most cases are in adolescents and young adultsC. Genotypes 1 and 2 are the cause of sporadic cases in industrialized countriesD. Waterborne outbreaks of HEV in developing countries may differ epidemiologically and genetically from cases reported in industrialized countries2. Based on the study by Dr. Bouquet and colleagues, which of the following statements about the genetic identity of HEV strains found in humans and swine during an 18-month period in France is most likely correct?A. About half of sequences belonged to genotype 3B. Similarity of about 75% was found between HEV sequences of human and swine originsC. Subtype 3c was the largest clusterD. Both human and swine populations had the same proportions of subtypes 3f, 3c, and 3e3. You are asked to consult with a public health department in southern France regarding a recent increase in autochthonous HEV cases. Based on the study by Dr. Bouquet and colleagues, which of the following statements is most likely to appear in your report?A. Spread of swine HEV infection within a herd occurs primarily by respiratory droplet transmissionB. The highest risk to humans eating pork products is associated with eating pork chopsC. Environmental factors are a more likely source of exposure than ingestion of pork productsD. A surveillance and control plan, either at the level of pig production or at the level of food processing, is recommendedActivity Evaluation

**Table:** 

1. The activity supported the learning objectives.				
Strongly Disagree				Strongly Agree
1	2	3	4	5
2. The material was organized clearly for learning to occur.				
Strongly Disagree				Strongly Agree
1	2	3	4	5
3. The content learned from this activity will impact my practice.				
Strongly Disagree				Strongly Agree
1	2	3	4	5
4. The activity was presented objectively and free of commercial bias.				
Strongly Disagree				Strongly Agree
1	2	3	4	5
